# Clinical Differences between COVID-19 and a COVID-Like Syndrome

**DOI:** 10.3390/jcm10112519

**Published:** 2021-06-07

**Authors:** Pierpaolo Di Micco, Giuseppe Camporese, Vincenzo Russo, Giuseppe Cardillo, Egidio Imbalzano, Antonella Tufano, Enrico Bernardi, Andrea Fontanella

**Affiliations:** 1 Department of Medicine, Buonconsiglio Fatebenefratelli Hospital of Naples, 80122 Naples, Italy; andreafontanella52@gmail.com; 2 Unit of Angiology, Department of Cardiac, Thoracic and Vascular Sciences, University Hospital of Padua, 35100 Padua, Italy; giuseppe.camporese@aopd.veneto.it; 3 Department of Translational Medical Sciences, University of Campania “Luigi Vanvitelli”, 80131 Naples, Italy; v.p.russo@libero.it; 4 Advanced Biochemistry Unit, Medylab, 80131 Naples, Italy; giuseppe.cardillo75@gmail.com; 5 Department of Clinical and Experimental Medicine, University of Messina, 98124 Messina, Italy; egidio.imbalzano@unime.it; 6 Internal Medicine, Federico II University of Naples, 80131 Naples, Italy; atufano@unina.it; 7 Department of Emergency and Accident Medicine, Treviso Civil Hospital, 31100 Treviso, Italy; enrico.bernardi@aulss2.veneto.it

**Keywords:** COVID-19, COVID-like syndrome, nasopharyngeal swab, SARS-CoV-2, atypical COVID-19 clinical presentation

## Abstract

COVID-19 is an infection due to SARS-CoV-2; this virus has been identified as the cause of the present pandemic. Several typical characteristics are present in this infection, in particular pneumonia with possible lung failure, but atypical clinical presentations are being described daily by physicians around the world. Ground-glass opacities with pneumonia are the most common and dangerous presentations of the COVID-19 disease, and they are usually associated with positive nasopharyngeal swab (NPS) tests with detectable SARS-CoV-2 viral RNA. Compared to the general population, hospital workers have been at a greater risk of infection ever since the first patients were hospitalized. However, hospital workers have also been reported as having COVID-like symptoms despite repeated negative swab tests but having tested positive for SARS-CoV-2 antibodies with serological tests. We can postulate that a COVID-like syndrome is possible, in particular in hospital workers, that is characterized by symptoms similar to those of COVID-19, but with repeated negative nasopharyngeal swabs. These repeated negative NSPs make the difference in daily clinical management with people that experienced a single false negative nasopharyngeal swab; furthermore, a clear clinical differentiation of these situations is still lacking in the literature. For this reason, here, we report our main findings from a cohort of patients with a COVID-like syndrome compared to a similar group affected by typical COVID-19.

## 1. Background

Seasonal flu and COVID-19 are infections characterized mainly by respiratory damage, sharing a similar set of symptoms, including fever, chills, malaise, cough, osteomyalgia, etc. COVID-19 spreads more efficiently and for a longer time-span than the flu, and—in particular—it can cause far more severe illnesses in some patients and can progress to pneumonia and (fatal) respiratory failure. Furthermore, COVID-19 is associated with long-standing and disabling respiratory symptoms when compared to the flu. Although we have gained a great deal of insights on COVID-19 since the pandemic began, further clinical presentations of this disease are described often, almost on a daily basis. Indeed, apart from classic respiratory syndrome, COVID-19 can frequently induce lymphopenia, a hypercoagulable state, and alterations to several laboratory markers, the latter being due to a prolonged presence of multifocal ground-glass opacities with pneumonia or the acute and subacute inflammation of other organs [1,2]. However, one of the most atypical clinical presentations of COVID-19 has been described among hospital workers since the first wave of SARS-CoV-2 infections [3]. These patients presented with COVID-like symptoms, such as fever, chills, and dyspnea; radiological findings that were suggestive of COVID-19 caused by the then-recent SARS-CoV-2 infection were also reported. Yet, constantly, these patients with COVID-like symptoms showed negative results after repeated nasopharyngeal swabs, which is the recognized test to detect the disease, while an increase in the serological markers of recent infection by SARS CoV2 (i.e., IgM antibodies) confirmed that the pathophysiology of the symptoms were due to SARS-CoV-2 infection and not other viral infections [3]. Based on these data, we could speculate that these findings may appear as an uncommon COVID-19, or as a co-infection of another atypical virus with SARS-CoV-2. Because a group of signs and symptoms was present and occurred in several cases, characterizing a particular abnormality or condition, we could define it as a specific syndrome (i.e., COVID-like syndrome) or as PCR-negative COVID-19; moreover, because of the presence of specific laboratory and radiological findings, we could perform a staging of the syndrome as can be done for typical COVID-19. Currently, PCR amplification of viral-RNA following a nasopharyngeal swab is considered the gold-standard to establish a diagnosis of symptomatic or asymptomatic COVID-19; however, major concerns have been raised concerning the likelihood of false-negative results in the community setting [4,5]. Hence, despite the presence of typical symptoms and of an increased risk of COVID-19 infection due to occupational exposure, both the identification and therapeutic management of this subgroup of subjects may be very difficult. This is of utmost importance, since the occurrence of PCR-negative COVID-19 in hospital workers may expose them, their colleagues, or family members, to an increased risk of contagion [3]. Furthermore, a delayed identification of the disease may also be associated with delayed treatment and possibly poorer outcomes [2]. Indeed, from a clinical point of view, the therapeutic approach as well as the outcome of patients with COVID-like syndrome remains uncertain. In this respect, some authors have already reported the usefulness of early treatment of patients highly suspected of having COVID-19, despite initially testing negative with nasopharyngeal swabs [6,7]. We report our clinical experience with a cohort of patients with COVID-like syndrome, and compared their clinical features and outcomes with a similar group of in-patients with a typical clinical presentation of COVID-19.

## 2. Patients and Methods

From 1 September 2020, to 31 December 2020, we identified 50 subjects with COVID-like syndrome who showed a recent onset of chills, fever, cough, myalgias, and asthenia, who had repeatedly tested negative with nasopharyngeal swabs but with a progressive increase of IgM and IgG anti-SARS-CoV-2 antibodies. The clinical characteristics and outcomes of these subjects were compared to those of 50 in-patients affected by COVID-19 with typical pneumonia with multifocal ground-glass opacities. The main clinical and laboratory signs, together with typical symptoms associated with COVID-19, such as chills, myalgias, fever, dyspnea, anosmia, increased C-reactive protein (CRP) and d-dimer, presence of lymphocytopenia, and evolution to lung failure and thrombotic events (e.g., deep vein thrombosis, lower limb ischemia, and stroke), were collected from both groups. The duration of symptoms and the association with pharmacological thromboprophylaxis with enoxaparin were also collected as the occurrence of bleedings. Laboratory markers were collected and compared between the two groups; threshold values were considered to be those commonly used for acute illness (Table 1). Furthermore, the basic clinical characteristics of both groups were also collected and are summarized in Table 2; age over 40 years, gender, ethnic background, presence of moderate obesity with BMI > 34.9, presence of more than 2 chronic cardiovascular and/or pneumological comorbidities, and hospitalization for an acute illness different from COVID-19 in last 30 days were considered.

Being mainly hospital workers, patients that referred to COVID-like symptoms had performed several routine serological tests against SARS-CoV-2, which were negative for IgM and/or IgG anti-SARS CoV2.

The clinical characteristics and outcomes of these subjects were compared to those of 50 in-patients, affected by COVID-19, with typical multifocal ground-glass opacities with pneumonia. 

Subjects with COVID-like symptoms were quarantined and underwent nasopharyngeal swabs every 3 to 4 days, from the beginning of symptoms up to the end of clinical manifestations. Starting from day 15 after the onset of symptoms, they also gave blood samples, to search for immunoglobulins against SARS-CoV-2, using the same schedule.

Real-time proteinase chain reaction (Light Cycler 480 II, ROCHE, Monza (MI), Italy) was used to detect SARS-CoV-2 viral-ribonucleic acid (v-RNA) from the nasopharyngeal swabs. Titration of antibodies against SARS-CoV-2 (IgM, and IgG) was obtained by ELISA chemiluminescence tests (Elecsys, Anti-SARS-CoV-2, Roche, Italy; LIAISON SARS-CoV-2, DiaSorin S.p.A., Vicenza, Italy). A number of laboratory tests, including d-dimer, C-reactive protein, and complete blood count, were performed for all patients.

All patients also underwent a chest CT-scan to evaluate the presence of pulmonary lesions, such as interstitial ground-glass opacities with pneumonia or other radiological manifestations of COVID-19; an ultrasound scan of the lower limbs was also performed to rule out deep- or superficial-vein thrombosis.

Statistical differences between categorical variables were tested for using the Fisher’s exact test. All statistical comparisons were performed using the statistical software package MATLAB R2016B.

## 3. Results

Outcomes and all clinical data are reported in Table 1. Myalgia was the most common symptom in patients with COVID-like syndrome, while weakness and anosmia were more frequent in patients with COVID-19.

No statistical differences were found in the demographic and basic characteristics of both groups (Table 2).

Moreover, no statistical differences were found in the incidence of cough, fever, and chills in the two groups. Similarly, d-dimer and C-reactive protein levels, as well as lymphocyte count, were substantially alike in both patient groups. In-patients with COVID-19 had significantly lower pulse oximetry readings than those with COVID-like syndrome. Thromboprophylaxis with enoxaparin was more frequently found in patients with COVID-19 than in patients with COVID-like syndrome. Deep- and superficial-vein thrombosis of the lower limbs, as well as major bleeding, were more common in the COVID-19 group than in the COVID-like syndrome group, although the difference was not statistically significant. Finally, ground-glass opacities with pneumonia, as detected by chest CT-scan, were significantly more frequent in patients with COVID-19 than in those with COVID-like syndrome.

## 4. Discussion

The Food and Drug Administration has indicated the possibility of the inaccuracy of diagnostic tests for suspected COVID-19. For instance, a sensitivity as low as 71% was reported for nasopharyngeal swabs by an early retrospective review from a community hospital in China [8]. False positive results erroneously label people as infected, causing unnecessary quarantine and contact tracing, while false negative results are more consequential, because infected subjects—who might be completely asymptomatic—are not isolated, thus potentially infecting many others.

There may be different explanations for false-negative results, including laboratory errors, but they usually occur within a single batch of tests and do appear sequentially in limited populations. Repeated negative swab results have been recorded in hospital workers with COVID-like symptoms [3].

Furthermore, being hospital workers, previous screenings of serological antibodies against SARS-CoV-2 (i.e., IgG or IgM) and/or nasopharyngeal swab (NPS) in the absence of symptoms, always tested negative.

A delayed identification of COVID-like syndrome may have relevant consequences for affected patients, in that they can get worse and unknowingly contaminate other people. Thus, given that the overall accuracy of real-time C-reactive protein for all clinical forms of SARS-CoV-2 infection is still being debated, it is suggested that patients at high suspicion of having COVID-19 with initially negative nasopharyngeal swab results undergo either bronchoalveolar lavage or chest CT-scan (to detect ground-glass opacities with pneumonia), or both, in order to speed up diagnosis and treatment. The role of false negative tests in this examination is debated, but for patients with COVID-like symptoms (i.e., COVID-like syndrome) the occurrence of repeated negative swabs is common [9].

The clinical approach to patients with repeated false negative nasopharyngeal swabs is particularly difficult, because the implications of a diagnostic and treatment delay are significantly worse than those of a single false negative test [3,10]. In fact, during the COVID-19 pandemic the occurrence of COVID-like syndrome in several subjects represented a relevant clinical concern. In this syndrome, the early onset of signs and symptoms, similar to those of COVID-19, is associated with laboratory and instrumental evidence of recent SARS-CoV-2 infection, namely the presence of antibodies against SARS-CoV-2 and the finding of ground-glass opacities with pneumonia. This is fundamental to establishing a correct diagnosis, to correctly isolate people in quarantine, and to adequately treat affected subjects, as the risk of viral transmission to other subjects is very high in cases of delayed diagnosis.

This is one of the first reports on the clinical evolution and outcomes of patients with COVID-like syndrome, which were observed and compared to a similar group of in-patients with typical COVID-19. As in other series of patients, hospital workers were more numerous in the out-patient cohort with COVID-like syndrome, than in the in-patient cohort with COVID-19 [3].

In this report, no substantial difference in the incidence of ground-glass opacities with pneumonia were observed between patients with COVID-19 and those with COVID-like syndrome, confirming that the pathological action of SARS-CoV-2 is specific to the lung. However, it is noteworthy that many more patients with COVID-19 had low pulse oximetry readings compared with those with COVID-like syndrome. In addition, no statistically significant differences were observed in terms of laboratory findings between patients with COVID-like syndrome and COVID-19. In particular, C-reactive protein, d-dimer levels, and lymphocyte count did not differ between groups. Similarly, high titers of IgM and IgG against SARS-CoV-2 were observed in both groups.

Along with ground-glass opacities with pneumonia, a hypercoagulable state and thrombotic complications have been frequently associated with COVID-19; venous thromboembolism being the more common type of thrombosis found in patients with COVID-19. Although the appropriate timing to screen in-patients with COVID-19 for deep vein thrombosis of the lower limbs is still a matter of debate [11,12], such an association impacts on the outcome of inpatients anyway [13,14,15,16,17,18,19] Then, in order to detect deep- or superficial-vein thrombosis, we choose to perform vascular imaging of the lower limbs also in patients with COVID-like syndrome.

Indeed, we recorded a greater number of thrombotic events in the in-patient cohort with COVID-19, although it was not statistically significant. The main clinical difference between patients with COVID-19 and COVID-like syndrome was represented by the presence, in the former group, of increased d-dimer levels and of other typical laboratory markers attesting to a hypercoagulable state, not associated with an increase of venous thrombotic events of the lower limbs. A really intriguing aspect is that the incidence of an objectively confirmed deep- or superficial-vein thrombosis of the lower limbs was higher in patients with typical COVID-19 than in those with COVID-like syndrome, despite thromboprophylaxis with low-molecular-weight heparin being routinely performed. Furthermore, another relevant clinical difference was the similar rate of major bleeding in both groups. On the other hand, in our study we were not able to record arterial events, such as ischemic stroke, transient ischemic attack, or critical limb ischemia, although the clinical association of arterial thrombosis and COVID-19 has been widely reported in other articles.

Of course, our study shows several limitations. First, someone may suggest that, in the case of typical symptoms of COVID-19 and a negative PCR after nasopharyngeal swab, the presence of SARS-CoV-2 could be detected from tracheal aspirate or similar pulmonary secretions; however, regarding this topic, we followed the guidelines from the Italian Ministry of Health for out-patients with COVID-19 [20]. Moreover, further reflection could be performed regarding the technical limitations of the swab procedure: COVID-19 patients with negative PCR might be COVID-19 patients with a low viral load or reduced colonization of the nasal epithelium, which could also explain the reduced incidence of anosmia in that group. Second, better clarification concerning a good outcome for this syndrome could be determined because it may depend on early diagnosis and treatment, as it was more frequently detected in hospital workers who are more exposed to periodic checks of infection and to their possible complications. The cohort of patients should be increased to a larger population and, for comparison, a better match regarding the clinical and demographic characteristics between patients with COVID-19 and patients with COVID-like syndrome should be performed. A real-life study or a clinical registry could be sufficient to provide this clinical information. Third, clinical outcomes of COVID-like syndrome could be better addressed, because we also found differences in the incidence of venous thrombotic events in terms of the incidence of major bleedings or the length of disease.

In conclusion, patients with COVID-like syndrome included in our study displayed a flu-like clinical presentation, including fever, cough, chills, myalgia, and weakness, frequently associated with ground-glass opacities with pneumonia, increased C-reactive protein levels, and lymphopenia. As compared with patients with COVID-19, patients with COVID-like syndrome had a statistically lower incidence of anosmia, while, from a prognostic point of view, the duration of symptoms was similar in both patient groups. However, we should speculate that this difference may be associated to several biases, such as the duration of hospitalization of patients affected by COVID-19 with the association to intense pharmacological treatments for hospitalized patients compared to those treated at home. Concerning deaths, only two cases were recorded in in-patients with COVID-19.

Being the first of a report series on patients affected by COVID-like syndrome, the current report provides several interesting clinical updates to the ongoing pandemic.

The use of nasopharyngeal swabs as the only gold standard for the identification of all forms of infections caused by SARS-CoV-2 may be associated with additional difficulties, different from the possibility of a single false negative test; in particular, patients with signs and symptoms similar to COVID-19 should be thoroughly evaluated from a clinical point of view, even if repeated swabs were negative. This is another limitation of the use of nasopharyngeal swabs as the only gold standard to detect SARS-CoV-2 infection [21]. COVID-like syndrome occurs more frequently in hospital workers, as previously reported in a case series report, and it does not require hospitalization in the majority of cases [3]. Moreover, COVID-like syndrome is less-frequently associated with complications, such as venous thromboembolism or bleeding, although the rate of pharmacological thromboprophylaxis with low-molecular-weight heparin is not frequent.

## Figures and Tables

**Table 1:** Clinical characteristics of patients with COVID-like syndrome and typical COVID-19.

Sign/Symptom	COVID-like Patients (*n* 50)	Typical COVID-19 (*n* 50)	*p*
Chills	41/50	39/50	0.7 *
Fever	42/50	36/50	0.2 *
Myalgia	46/50	35/50	5.6 × 10^−3^ **
Headache	38/50	31/50	0.1 *
Weakness	45/50	50/50	0.02 **
Anosmia	14/50	44/50	3.6 × 10^−10^ **
Lymphopenia (<1500 mmcube)	49/50	47/50	0.4 *
Increased CRP (>5 mg/dL)	47/50	50/50	0.09 *
Increased d-dimer (>500 µg/dL)	36/50	43/50	0.09 *
sO2 < 93%	2/50	46/50	1.4 × 10^−21^ **
Deep or superficial vein thrombosis	0/50	3/50	0.09 *
Major bleeding	1/50	2/50	0.7 *
Thromboprophylaxis with enoxaparin	19/50	50/50	2.9 × 10^−12^ **
Ischemic stroke or transient ischemic attack	0/50	0/50	1 *
Critical limb ischemia	0/50	0/50	1 *
Pneumonia with ground-glass opacities at chest CT-scan	36/50	50/50	4.8 × 10^−5^ **
Symptoms lasting > 14 days	22/50	23/50	0.09 *
IgM/IgG anti-SARS-CoV2	50/50	50/50	1.00 *

* not statistically significant; ** statistically significant; CRP, C-reactive protein; sO2, oxygen saturation; IgM, immunoglobulin M; IgG, immunoglobulin G; CT, computerized tomography.

**Table 2:** Clinical and demographic anamnestic characteristics of analyzed patients.

Demographic and Anamnestic Characteristics	COVID-Like Syndrome (*n* 50)	COVID-19 (*n* 50)	*p*
Age > 40 y	23/50	22/50	0.09, ns *
Gender, male	36/50	42/50	0.09, ns *
Caucasian ethnic group	50/50	50/50	0.09, ns *
Moderate obesity with BMI > 34.9	4/50	6/50	0.3, ns *
Presence of more than 2 chronic cardiovascular or pneumological comorbidities	14/50	16/50	0.09, ns *
Recent hospitalization for acute illness different from COVID-19 in last 2 months	0/50	0/50	1, ns *

* not statistically significant; BMI, body mass index.
