# Correction: Di Micco et al. Clinical Differences between COVID-19 and a COVID-like Syndrome. *J. Clin. Med.* 2021, *10*, 2519

**DOI:** 10.3390/jcm11113076

**Published:** 2022-05-30

**Authors:** Pierpaolo Di Micco, Giuseppe Camporese, Vincenzo Russo, Giuseppe Cardillo, Egidio Imbalzano, Antonella Tufano, Enrico Bernardi, Andrea Fontanella

**Affiliations:** 1 Department of Medicine, Buonconsiglio Fatebenefratelli Hospital of Naples, 80122 Naples, Italy; andreafontanella52@gmail.com; 2 Unit of Angiology, Department of Cardiac, Thoracic and Vascular Sciences, University Hospital of Padua, 35100 Padua, Italy; giuseppe.camporese@aopd.veneto.it; 3 Department of Translational Medical Sciences, University of Campania “Luigi Vanvitelli”, 80131 Naples, Italy; v.p.russo@libero.it; 4 Advanced Biochemistry Unit, Medylab, 80131 Naples, Italy; giuseppe.cardillo75@gmail.com; 5 Department of Clinical and Experimental Medicine, University of Messina, 98124 Messina, Italy; egidio.imbalzano@unime.it; 6 Internal Medicine, Federico II University of Naples, 80131 Naples, Italy; atufano@unina.it; 7 Department of Emergency and Accident Medicine, Treviso Civil Hospital, 31100 Treviso, Italy; enrico.bernardi@aulss2.veneto.it

**Incorrect Affiliation**

In the published article [1], there was an error in the following affiliation: “Department of Translational Medical Sciences, Monaldi Hospital, University of Campania “Luigi Vanvitelli”, 80131 Naples, Italy”, it should have been “Department of Translational Medical Sciences, University of Campania “Luigi Vanvitelli”, 80131 Naples, Italy”.

**Error in Table**

In the original published article [1], there was a mistake in Table 1. The indexes in the scientific notation are missing. The corrected version appears below.

**Table 1:** Clinical characteristics of patients with COVID-19-like syndrome and typical COVID-19.

Sign/Symptom	COVID-like Patients (*n* 50)	Typical COVID-19 (*n* 50)	*p*
Chills	41/50	39/50	0.7 *
Fever	42/50	36/50	0.2 *
Myalgia	46/50	35/50	5.6 × 10^−3^ **
Headache	38/50	31/50	0.1 *
Weakness	45/50	50/50	0.02 **
Anosmia	14/50	44/50	3.6 × 10^−10^ **
Lymphopenia (<1500 mmcube)	49/50	47/50	0.4 *
Increased CRP (>5 mg/dL)	47/50	50/50	0.09 *
Increased d-dimer (>500 µg/dL)	36/50	43/50	0.09 *
sO2 < 93%	2/50	46/50	1.4 × 10^−21^ **
Deep or superficial vein thrombosis	0/50	3/50	0.09 *
Major bleeding	1/50	2/50	0.7 *
Thromboprophylaxis with enoxaparin	19/50	50/50	2.9 × 10^−12^ **
Ischemic stroke or transient ischemic attack	0/50	0/50	1 *
Critical limb ischemia	0/50	0/50	1 *
Pneumonia with ground-glass opacities at chest CT-scan	36/50	50/50	4.8 × 10^−5^ **
Symptoms lasting > 14 days	22/50	23/50	0.09 *
IgM/IgG anti-SARS-CoV2	50/50	50/50	1.00 *

* not statistically significant; ** statistically significant; CRP, C-reactive protein; sO2, oxygen saturation; IgM, immunoglobulin M; IgG, immunoglobulin G; CT, computerized tomography.

The authors apologize for any inconvenience that may have been caused, and state that the scientific conclusions are unaffected. The original article has been updated.
